# ﻿*Cirrhilabrusfinifenmaa* (Teleostei, Labridae), a new species of fairy wrasse from the Maldives, with comments on the taxonomic identity of *C.rubrisquamis* and *C.wakanda*

**DOI:** 10.3897/zookeys.1088.78139

**Published:** 2022-03-08

**Authors:** Yi-Kai Tea, Ahmed Najeeb, Joseph Rowlett, Luiz A. Rocha

**Affiliations:** 1 School of Life and Environmental Sciences, University of Sydney, Sydney, Australia University of Sydney Sydney Australia; 2 Ichthyology, Australian Museum Research Institute, 1 William Street, Sydney, New South Wales 2010, Australia Australian Museum Research Institute Sydney Australia; 3 Maldives Marine Research Institute, Ministry of Fisheries Marine Resources and Agriculture, Malé 20025, Maldives Maldives Marine Research Institute Malé Maldives; 4 Field Museum of Natural History, Chicago, Illinois, 60605, USA Field Museum of Natural History Chicago United States of America; 5 Department of ichthyology, California Academy of Sciences, San Francisco, California 94118, USA California Academy of Sciences San Francisco United States of America

**Keywords:** Coral reefs, deep reefs, Indian Ocean, mesophotic, reef fish

## Abstract

*Cirrhilabrusrubrisquamis* is redescribed on the basis of the juvenile holotype and compared to known species of *Cirrhilabrus*. Examination of material from the Maldives identified as *C.rubrisquamis* reveal differences from the holotype collected from the Chagos Archipelago. Consequently, the Maldivian specimens are herein described as *Cirrhilabrusfinifenmaa***sp. nov.**, on the basis of the holotype and twelve paratypes. The new species differs from all congeners in having: males with anterior third to half of body bright magenta, peach to orange-pink posteriorly; lateral line with 22–26 pored scales (16–18 in the dorso-anterior series, 6–8 in the posterior peduncular series); tenth to eleventh dorsal-fin spine longest (14.0–15.5% SL); scales on the opercle, chest, isthmus, and anterior third of the body with a dark purple-red central region (purple in alcohol), the markings joining appearing crosshatched; dorsal, caudal, anal, and pelvic-fin rays purple in alcohol. Meristic details and coloration patterns of *C.rubrisquamis* are very similar to *C.wakanda* from Tanzania, Africa, although synonymy of both species cannot be determined without additional material from Chagos. This potential synonymy is briefly discussed; however, until such material becomes available, the taxonomic statuses of *C.wakanda* and *C.rubrisquamis* are here provisionally regarded as valid.

## ﻿Introduction

Randall and Emery (1983) described *Cirrhilabrusrubrisquamis* from the 40.6 mm standard length (SL) holotype collected at 41–48 m depth in Isla Fouquet, Peros Banhos Atoll, Chagos Archipelago. Their description was based on a single juvenile specimen, and so appropriate details regarding adult coloration and characters were unavailable. In his subsequent review of *Cirrhilabrus* from the Western Indian Ocean, Randall (1995) rediagnosed *C.rubrisquamis* with additional material from Vilingili Island, North Malé Atoll, Maldives, thus expanding its distribution, and stating that “it is satisfying to be able to diagnose and illustrate the adult female and male of this colorful species”. Although Randall’s specimens consisted mostly of mature individuals of both sexes, it also included a juvenile that differed in coloration pattern from the similarly-sized holotype from Chagos, thus raising the question as to whether the specimens from the Maldives represent a different species. Re-examination of the aforementioned Maldivian specimens indicate that they indeed represent a different species from the nominal Chagossian *C.rubrisquamis*. To resolve this taxonomic conundrum, the Maldivian specimens are examined and compared with *C.rubrisquamis* sensu stricto and related congeners, and here described as the new species *Cirrhilabrusfinifenmaa*. The holotype of *C.rubrisquamis* is redescribed and compared to related *Cirrhilabrus*. It is shown to be a valid species of the genus and a possible senior synonym of *C.wakanda*. We briefly comment on the taxonomic status of both species, but provisionally regard both as valid pending additional material of adult *C.rubrisquamis* from Chagos.

## ﻿Materials and methods

Methods for counting and measuring mostly follow Randall and Masuda (1991). Gill rakers are presented as upper (epibranchial) + lower (ceratobranchial); the angle raker is included in the second count. Counts of lateral-line scales are given in two parts, the dorso-anterior series and the midlateral peduncular series. The latter series consists of a larger pored scale overlapping the caudal-fin base, which we include in the count. In the description that follows, data are presented first for the holotype, followed by the range of minimum–maximum values of the paratypes in parentheses where different. Where counts were recorded bilaterally, both counts are given and separated from each other by a slash; the first count presented is the left count. Morphometric values are expressed as percentage of standard length (Table 1). Osteological details were taken from radiographs of the holotype and paratypes. Institutional codes follow Sabaj (2020) and are as follows: Australian Museum, Sydney (**AMS**); Bernice Pauahi Bishop Museum (**BPBM**); Royal Ontario Museum (**ROM**); California Academy of Sciences (**CAS**); Zoological Reference Collection of the Lee Kong Chian Natural History Museum at the National University of Singapore (**ZRC**).

**Table 1.** Proportional measurements for holotype of *Cirrhilabrusrubrisquamis*, and type series of *C.finifenmaa* sp. nov., and *C.wakanda*, expressed as percentage of the standard length. Data for *C.wakanda* (*n* = 5) summarized from Tea et al. (2019).

	*Cirrhilabrusrubrisquamis*	*Cirrhilabrusfinifenmaa sp. nov.*													*Cirrhilabruswakanda*
	ROM 35932	BPBM 33094	BPBM 41385 (formerly BPBM 33094)					ZRC 62259	ZRC 62249	ZRC 61604	AMS I.50058-001			CAS-ICH 247311 (ex AMS I.50058-001)	CAS 246395– CAS 246399
	Holotype	Holotype	Paratypes												Holotype and four paratypes
Sex	Juvenile	Male	Male	Male	Female	Female	Juvenile	Male	Male	Female	Male	Male	Female	Female	2 Males and three females
Standard length (mm)	40.6	69.2	70.5	57.6	52.9	52.1	35.8	76.7	54.2	47.9	69.1	59.7	54.0	57.2	54.3–70.3
Body depth	28.8	31.6	30.2	32.3	32.3	29.8	35.8	31.3	28.2	29.0	31.4	31.2	28.3	29.4	29.8–31.9
Body width	13.3	13.9	11.8	13.2	14.2	13.2	12.6	14.6	13.1	12.1	17.2	16.1	15.2	15.7	11.8–14.5
Head length	35.0	31.6	30.4	31.3	32.1	32.4	33.2	31.0	33.4	35.9	33.9	33.5	32.6	33.0	27.7–31.2
Snout length	9.6	9.5	8.8	9.0	8.1	8.1	9.5	8.5	8.5	10.2	9.1	8.4	8.1	8.4	7.4–8.9
Orbit diameter	10.1	8.8	9.5	9.2	10.2	10.2	11.7	9.6	10.3	11.1	9.0	9.7	10.0	9.1	6.6–9.0
Interorbital width	7.4	9.1	8.2	9.5	9.8	9.4	9.5	8.9	9.2	9.0	9.3	10.2	9.4	10.0	7.7–9.6
Upper jaw length	6.4	7.4	5.8	8.7	7.0	7.9	8.1	9.3	5.7	7.3	5.8	5.7	4.6	4.2	6.5–8.3
Caudal-peduncle depth	16.5	14.5	15.5	15.1	15.9	15.9	15.4	14.3	15.9	16.3	17.7	16.9	14.8	15.4	14.8–16.5
Caudal-peduncle length	13.5	15.8	17.9	16.5	15.9	15.2	10.1	18.9	11.8	14.8	15.1	15.1	13.1	14.3	12.8–16.5
Predorsal length	36.9	31.2	31.9	33.3	31.6	35.9	34.4	33.4	34.9	38.4	33.6	34.0	33.0	34.1	31.7–33.8
Preanal length	65.3	61.4	61.0	60.1	67.3	61.8	65.6	55.7	58.1	61.8	57.6	59.6	61.7	61.0	58.5–61.4
Prepelvic length	40.9	35.7	35.7	34.7	34.4	34.9	38.3	33.5	34.3	38.6	35.0	34.5	34.6	33.9	31.5–36.5
Dorsal-fin base	57.4	60.4	57.7	60.8	60.7	58.0	57.5	61.3	58.5	61.4	60.0	61.0	57.6	61.0	55.3–63.2
First dorsal spine	9.1	7.9	6.8	8.0	7.4	7.5	7.5	6.8	7.0	7.3	5.6	6.7	7.4	6.8	5.2–6.5
Longest dorsal spine	15.0	15.5	15.0	15.1	15.5	15.2	14.0	15.6	15.3	15.9	10.6	14.2	15.6	14.3	11.9–14.3
Longest dorsal ray	17.2	19.1	20.1	18.1	18.1	17.7	damaged	21.4	15.3	20.0	21.7	17.1	18.1	18.7	16.7–19.0
Anal-fin base	22.4	28.0	24.5	24.8	25.7	22.8	25.1	26.6	26.0	24.0	28.5	25.6	27.0	26.9	25.3–27.6
First anal spine	8.9	6.4	7.4	6.4	6.4	5.8	5.9	6.0	6.1	6.5	6.4	4.9	8.3	6.6	5.2–6.4
Second anal spine	13.5	9.7	10.9	10.8	11.0	11.3	10.6	11.0	12.4	10.2	11.1	9.7	11.5	10.1	9.1–10.1
Third anal spine	14.3	11.6	11.2	11.8	12.1	13.2	11.7	12.3	12.5	10.2	13.0	11.7	12.2	11.4	10.5–11.4
Longest anal ray	18.0	20.2	19.7	17.0	17.0	16.9	14.8	23.3	20.5	21.1	21.7	20.1	16.9	18.4	14.5–17.9
Caudal-fin length	29.6	27.5	29.8	25.9	28.0	28.8	32.1	30.0	33.6	30.7	28.5	29.8	29.6	32.9	25.4–31.6
Pectoral-fin length	19.7	20.2	20.0	21.0	21.4	20.9	19.3	23.5	19.7	24.0	6.4	21.8	20.7	21.5	18.3–21.8
Pelvic-spine length	14.8	11.3	11.5	12.2	12.7	13.4	13.4	11.5	12.0	12.9	11.1	11.4	11.9	11.0	11.0–12.1
Pelvic-fin length	19.2	22.7	17.2	18.8	20.8	19.2	20.4	22.9	18.6	18.4	13.0	16.8	17.8	21.7	15.5–18.8

## ﻿Taxonomy

### ﻿ Cirrhilabrus rubrisquamis

Taxon classificationAnimaliaPerciformesLabridae

Randall & Emery, 1983

37DB3724-992F-59AE-B456-A72F03412E9A

**Figure 1.**
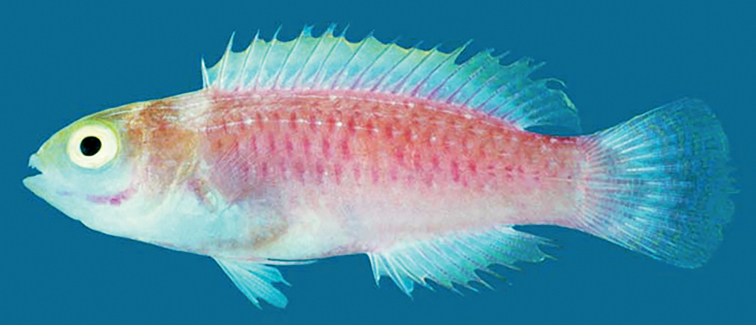
*Cirrhilabrusrubrisquamis*, ROM 35932, 40.6 mm SL, juvenile holotype, Isla Fouquet, Peros Banhos Atoll, Chagos Archipelago. Photograph by AR Emery and R Winterbottom.

Rosy-scaled Fairy Wrasse Fig. 1 , Table 1

Cirrhilabrus rubrisquamis Randall & Emery, 1983: 21; fig. 1 (description); Winterbottom et al. 1989: 53; pl VII-E (checklist, fishes of the Chagos Archipelago); Winterbottom and Anderson 1997: 15 (revised checklist, fishes of the Chagos Archipelago)

#### Holotype.

*Cirrhilabrusrubrisquamis*: ROM 35932, 40.6 mm SL, juvenile, Isla Fouquet, Peros Banhos Atoll, Chagos Archipelago (5°21'3"S, 71°48'57"E).

#### Diagnosis.

*Cirrhilabrusrubrisquamis* shares similar meristic characters to other members of this genus, in particular *C.wakanda* (the potential synonymy of both species is discussed below). However, the holotype is readily distinguished from related congeners in having the following combination of characters: lateral line with 21 or 22 pored scales (15 or 16 in the dorso-anterior series, six in the posterior peduncular series); gill rakers 16; caudal fin round with blue and yellow vermiculation in life; dorsal two-thirds of body with purple scales arranged in a chain-link pattern in life.

#### Description.

Dorsal-fin rays XI,9; all soft rays branched except first; anal-fin rays III,9; all soft rays branched except first; last dorsal and anal-fin ray branched to base; pectoral-fin rays 15/15, upper two unbranched; pelvic-fin rays I,5; principal caudal-fin rays 7+6, uppermost and lowermost unbranched; upper procurrent caudal-fin rays 5, lower procurrent caudal-fin rays 5; lateral line interrupted, with dorso-anterior series of pored scales 16/15 and midlateral posterior peduncular series 6/6; first pored scale on posterior peduncular series often pitted; last pored scale on posterior peduncular series enlarged and overlapping hypural crease; scales above lateral line to origin of dorsal fin 2; scales below lateral line to origin of anal fin 6; median predorsal scales 5; median prepelvic scales 5; rows of scales on cheek 2; circumpeduncular scales 16; gill rakers 5 + 11 = 16; pseudobranchial filament count not made, owing to small size of specimen; vertebrae 9 + 16; epineurals 12.

Body moderately elongate and compressed, depth 3.5 in SL, width 2.2 in depth; head length (HL) 2.9 in SL; snout pointed, its length 3.6 in HL; orbit diameter 3.5 in HL; depth of caudal peduncle 2.1 in HL. Mouth small, terminal, and oblique, with maxilla almost reaching vertical at front edge of orbit; dentition typical of genus with three pairs of canine teeth present anteriorly at side of upper jaw, first forward-projecting, next two strongly recurved and outcurved, third longest; an irregular row of very small conical teeth medial to upper canines; lower jaw with a single stout pair of canines anteriorly which protrude obliquely outward and are slightly lateral to medial pair of upper jaw; no teeth on roof of mouth.

Posterior margin of preopercle with 29/27 very fine serrations; margins of posterior and ventral edges of preopercle free to about level of middle pupil. Anterior nostril in short membranous tube, located nearer to orbit than snout tip; posterior nostril larger, roughly ovoid to rectangular, located just medial and anterior to upper edge of eye. Scales cycloid; head scaled except snout and interorbital space; six large scales on opercle; a broad naked zone on membranous edge of preopercle; a row of large, elongate, pointed scales along base of dorsal fin, one per element, scales progressively shorter posteriorly on soft portion of fin; anal fin with a similar basal row of scales; last pored scale of lateral line (posterior to hypural plate) enlarged and pointed; one scale above and below last pored scale also enlarged; pectoral fins naked except for a few small scales at extreme base; a single large scale at base of each pelvic fin, about three-fourths length of pelvic spine.

Origin of dorsal fin above second or third lateral-line scale, predorsal length 2.7 in SL; first 1–5 dorsal-fin spines progressively longer, sixth to tenth subequal, eleventh longest, 2.3 in HL; interspinous membranes of dorsal fin in males extend beyond dorsal-fin spines, with each membrane extending in a pointed cirri beyond spine; eighth dorsal-fin soft ray longest, 2.0 in HL, remaining rays progressively shorter; origin of anal fin below base of tenth dorsal-fin spine; third anal-fin spine longest, 2.4 in HL; interspinous membranes of anal fin extended as on dorsal fin; anal-fin soft rays relatively uniform in length, eighth longest, 1.9 in HL; dorsal and anal-fin rays just reaching caudal-fin base; caudal fin rounded; pectoral fins short, reaching vertical between bases of fourth or fifth dorsal-fin spines, longest ray 1.8 in HL; origin of pelvic fins below lower base of pectoral fins; pelvic fins short, not reaching past anal fin origin, longest ray 1.8 in HL.

#### Coloration of holotype in life.

Based on color photograph of holotype when fresh (Fig. 1): head yellow, brownish posteriorly; lower part of head whitish to pale pink; preopercle prominently purple on outer edge; iris yellow; body orangey pink, fading to whitish pink ventrally; body with a network of dark purple scales arranged in a chain link pattern from just after dorsal fin origin to edge of caudal peduncle, absent from lower third of body; dorsal fin hyaline, yellow on distal half; posterior dorsal fin yellowish hyaline with metallic blue spots; caudal fin bluish hyaline with yellow and blue vermiculation, often broken in spots; anal fin similar to dorsal fin; pelvic fins hyaline; pectoral fin base with a purple band; pectoral fins pinkish hyaline.

#### Coloration of holotype in alcohol.

Uniformly pale tan, median and paired fins translucent hyaline. No evidence of purple scale markings, likely due to immaturity and/or loss of coloration over time.

### ﻿ Cirrhilabrus finifenmaa sp. nov.

Taxon classificationAnimaliaPerciformesLabridae

0B775A51-EC16-5786-A353-BC0757D00FE3

http://zoobank.org/E6D891D1-FAB0-45B6-BE5A-D4123BF85A13

**Figure 2.**
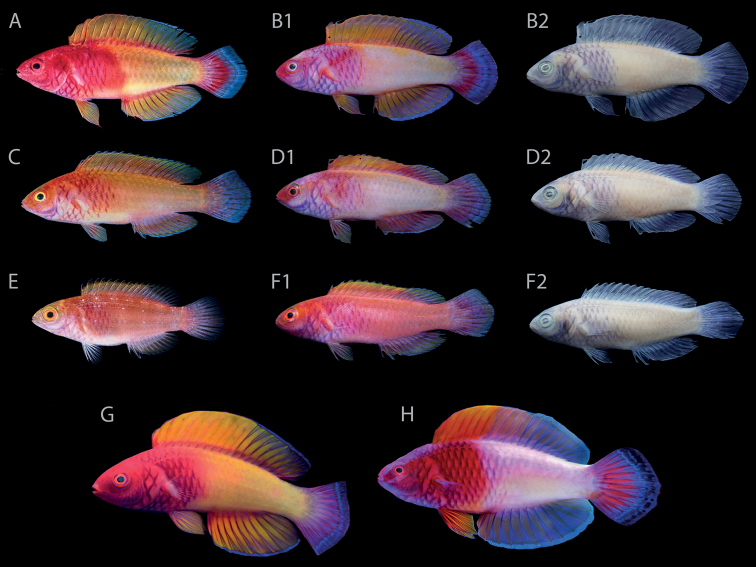
*Cirrhilabrusfinifenmaa*, sp. nov., not to scale **A** male holotype, BPBM 33094, 69.2 mm SL, Vilingili Island, North Malé Atoll, Maldives **B1, B2** male paratype, AMS I.50058-001, 69.1 mm SL, Hulhumalé Island, North Malé Atoll, Maldives, in life and in preservation respectively **C** young male paratype, BPBM 41385 (formerly BPBM 33094), 57.6 mm SL, same data as holotype **D1, D2** young male paratype, AMS I.50058-001, 59.7 mm SL, Hulhumalé Island, North Malé Atoll, Maldives, in life and in preservation respectively **E** juvenile paratype, BPBM 41385 (formerly BPBM 33094), 35.8 mm SL, same data as holotype **F1, F2** female paratype, AMS I.50058-001, 54.0 mm SL, Hulhumalé Island, North Malé Atoll, Maldives, in life and in preservation respectively **G** male paratype in life, ZRC 62259, 76.7 mm SL, aquarium specimen from Maldives **H** male in nuptial colors, aquarium specimen from Maldives, specimen not retained. Photographs by the late JE Randall (**A, C, E**), AN (**B1–B2, D1–D2, F1–F2**), and YKT (**G, H**).

**Figure 3.**
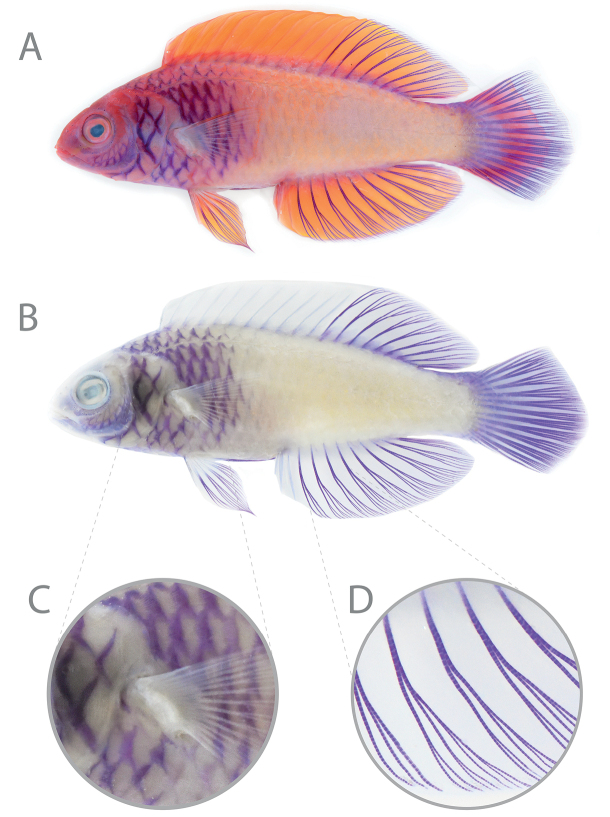
*Cirrhilabrusfinifenmaa* sp. nov., male paratype, ZRC 62259, 76.7 mm SL, (**A**) freshly euthanized (**B**) and in preservation. Note (**C**) purple scale markings forming a crosshatched pattern and (**D**) purple fin rays in preservation. Photographs by YKT.

**Figure 4.**
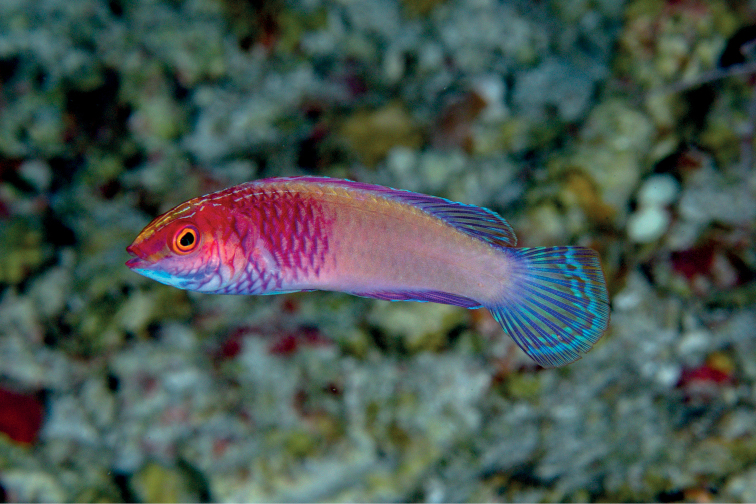
*Cirrhilabrusfinifenmaa* sp. nov., underwater photograph from Rasdhoo Atoll, Maldives, 60 m. Photograph by LAR.

Rose-Veiled Fairy Wrasse Figs 2 , 3 , 4 ; Table 1

Cirrhilabrus rubrisquamis (non Randall & Emery, 1983): Randall and Anderson, 1993: pl. 6C (checklist, underwater photograph from Maldives); Randall 1995: figs 5–7 (preserved specimens, BPBM 33094); Hawkins et al. 2000: 85 (IUCN assessment); Allen et al. 2008: (in part [Maldivian distribution], checklist of valid species of Cirrhilabrus); Kuiter 2010: 135 (color photographs A–C; aquarium specimens from Maldives); Allen et al. 2015: (in part [Maldivian distribution], checklist of valid species of Cirrhilabrus); Hawkins et al. 2016: fig. 1 (fluorescence spectrometry of a male specimen); Tea and Gill 2017: fig. 8H (color photograph of an aquarium specimen from Maldives); Tea et al. 2018: fig. 10 (preserved specimen, BPBM 33094); Tea et al. 2019: fig. 5D (color photograph of an aquarium specimen from Maldives); Tea et al. 2021a (included as part of a phylogenomic study of the genus).

#### Holotype.

BPBM 33094, 69.2 mm SL, male, east coast of Vilingili Island, North Malé Atoll, Maldives, 52 m, collected by John E. Randall with quinaldine and spear, 23–25 March 1988 (Fig. 2A).

#### Paratypes.

BPBM 41385 (formerly BPBM 33094) (5), 35.8–70.5 mm SL, 1 juvenile, 2 females, 2 males, collected with male holotype (Fig. 2C, E); AMS I.50058-001 (3), 54.0–69.1 mm SL, 2 males, 1 female, Hulhumalé Island, North Malé Atoll, Maldives (4°14'6.66"N, 73°33'15.47"E), 35–40 m, collected by Ibrahim Rasheed with hand nets, 15 August 2021 (Fig. 2B1–B2, D1–D2, F1–F2); CAS-ICH 247311 (ex AMS I.50058-001), 57.2 mm SL, female, collected with AMS I.50058-001; ZRC 62259, 76.7 mm SL, male, aquarium specimen from Maldives (Figs 2G, 3); ZRC 62249, 54.2 mm SL, male, aquarium specimen from Maldives; ZRC 61604, 47.9 mm SL, female, aquarium specimen from Maldives.

#### Diagnosis.

A species of *Cirrhilabrus* distinguished from congeners based on the following combination of characters: males with anterior third to half of body bright magenta, peach to orange-pink posteriorly; lateral line with 22–26 pored scales (16–18 in the dorso-anterior series, 6–8 in the posterior peduncular series); tenth to eleventh dorsal-fin spine longest (14.0–15.5% SL); scales on the opercle, chest, isthmus, and anterior third of the body with a dark purple-red central region, the markings joining to form a crosshatched appearance (purple in alcohol); dorsal, caudal, anal, and pelvic-fin rays purple in alcohol.

#### Description.

Dorsal-fin rays XI,9; all soft rays branched except first; anal-fin rays III,9; all soft rays branched except first; last dorsal and anal-fin ray branched to base; pectoral-fin rays 15 (right side removed in all three ZRC paratypes), upper two unbranched; pelvic-fin rays I,5; principal caudal-fin rays 7+6, uppermost and lowermost unbranched; upper procurrent caudal-fin rays 6 (6–7), lower procurrent caudal-fin rays 6 (5–6); lateral line interrupted, with dorso-anterior series of pored scales 17/17 (16–18) and midlateral posterior peduncular series 8/7 (6–8); first pored scale on posterior peduncular series often pitted; last pored scale on posterior peduncular series enlarged and overlapping hypural crease; scales above lateral line to origin of dorsal fin 2; scales below lateral line to origin of anal fin 6 (6–7); median predorsal scales 5 (4–5); median prepelvic scales 6 (5–6); rows of scales on cheek 2; circumpeduncular scales 14 (14–16); gill rakers 5 (5–6) + 11 (9–12) = 16 (14–18); pseudobranchial filaments 11 (10–11); vertebrae 9 + 16; epineurals 13.

Body moderately elongate and compressed, depth 3.2 (2.8–3.5) in SL, width 2.3 (1.8–2.8) in depth; head length (HL) 3.2 (2.8–3.3) in SL; snout pointed, its length 3.3 (3.3–4.0) in HL; orbit diameter 3.6 (2.8–3.8) in HL; depth of caudal peduncle 2.2 (1.9–2.2) in HL. Mouth small, terminal, and oblique, with maxilla almost reaching vertical at front edge of orbit; dentition typical of genus with three pairs of canine teeth present anteriorly at side of upper jaw, first forward-projecting, next two strongly recurved and outcurved, third longest; an irregular row of very small conical teeth medial to upper canines; lower jaw with a single stout pair of canines anteriorly which protrude obliquely outward and are slightly lateral to medial pair of upper jaw; no teeth on roof of mouth.

Posterior margin of preopercle with 35/36 (27–39) very fine serrations; margins of posterior and ventral edges of preopercle free to about level of middle pupil. Anterior nostril in short membranous tube, located nearer to orbit than snout tip; posterior nostril larger, roughly ovoid to rectangular, located just medial and anterior to upper edge of eye. Scales cycloid; head scaled except snout and interorbital space; 6 (6–7) large scales on opercle; a broad naked zone on membranous edge of preopercle; a row of large, elongate, pointed scales along base of dorsal fin, one per element, scales progressively shorter posteriorly on soft portion of fin; anal fin with a similar basal row of scales; last pored scale of lateral line (posterior to hypural plate) enlarged and pointed; one scale above and below last pored scale also enlarged; pectoral fins naked except for a few small scales at extreme base; a single large scale at base of each pelvic fin, about three-fourths length of pelvic spine.

Origin of dorsal fin above second or third lateral-line scale, predorsal length 3.2 (2.6–3.2) in SL; first 1–5 dorsal-fin spines progressively longer, sixth to ninth subequal, tenth to eleventh longest, 2.0 (2.0–2.4) in HL; interspinous membranes of dorsal fin in males extend beyond dorsal-fin spines, with each membrane extending in a pointed cirri beyond spine; eighth to ninth dorsal-fin soft ray longest (three paratypes with second dorsal-fin soft ray longest), 1.6 (1.5–2.3) in HL, remaining rays progressively shorter; origin of anal fin below base of 10^th^ dorsal-fin spine; third anal-fin spine longest, 2.7 (2.4–3.5) in HL; interspinous membranes of anal fin extended as on dorsal fin; anal-fin soft rays relatively uniform in length, seventh to ninth longest, 1.6 (1.5–2.2) in HL; dorsal and anal-fin rays just reaching past caudal-fin base; caudal fin rounded; pectoral fins short, reaching vertical between bases of 6^th^ or seventh dorsal-fin spines, longest ray 1.6 (1.3–1.7) in HL; origin of pelvic fins below lower base of pectoral fins; pelvic fins short, not reaching past anal fin origin, longest ray 1.4 (1.4–2.0) in HL.

#### Coloration of males in life.

Based on color photograph of holotype and paratypes when fresh, and live specimens photographed in the field and aquaria (Figs 2A, B1, C, D1, G, H, 3A, 4): head magenta to orange-red, red stripe present from mid-upper lip to mid-upper edge of orbit, continuing to anterior half of dorsal fin base; second stripe of similar color present from lower edge of maxilla to mid-lower edge of orbit; nape to predorsal region with four to five thin white stripes; preopercle prominently purple on outer edge; iris bright yellow with red ring around pupil; anterior third of body magenta to orange-red pink, remaining two thirds peach to orange-pink; scales on opercle, chest, isthmus and anterior third of body with dark purple-red central region, markings joining to form a crosshatched appearance; dorsal fin orange, sometimes flush with pink, becoming progressively hyaline posteriorly; outer edge of dorsal fin metallic blue; segmented dorsal-fin rays deep purple; basal two-thirds of caudal fin magenta to rose-red, remaining third metallic blue; caudal-fin rays deep purple; anal fin similar to dorsal fin; pelvic fins translucent orange, rays deep purple.

#### Coloration of females and juveniles in life.

Based on color photographs of holotype and paratypes when freshly dead, and live specimens photographed in the field and aquaria (Fig. 2E, F1): similar to male, except distinction between magenta head and anterior body from paler posterior two-thirds less prominent, sometimes suffused; coloration on median fins less obvious, appearing hyaline or pale orange-pink; juveniles similar to females, except uniformly suffused-pink; median fins pinkish hyaline.

#### Coloration in alcohol.

Similar to coloration in life, except head and body light tan to cream; edge of preopercle purple; dark purple-red scale markings now purple; dentary, Angulo-articular, and bony edge of preopercle purple; segmented rays of dorsal, anal, caudal, and pelvic fins purple (Figs 2B2, D2, F2, 3B–D).

#### Etymology.

The epithet is from the Dhivehi "finifenmaa", meaning rose, alluding to the live coloration of this species. The pink rose fiyatoshi finifenmaa (*Rosa* spp.) is also the national flower of the Maldives. To be treated as a noun in apposition. The common name is given after the facial patterns of the species.

#### Habitat and distribution.

*Cirrhilabrusfinifenmaa* is presently known from Maldives and Sri Lanka, at depths ranging from 40–70 m (Fig. 4). The species belongs to the *C.jordani* species complex, a group of deep-water fairy wrasses found mostly in mesophotic coral ecosystems. It is likely that this species occurs in greater depths. Like other species of *Cirrhilabrus*, *C.finifenmaa* frequents rubble bottoms scattered with loose coral cover.

#### Remarks.

Mitochondrial COI and 16S sequences for *C.finifenmaa* are publicly available on GenBank (accession numbers MH780161 and MH780159 respectively; previously identified and listed as *C.rubrisquamis*). We exercise caution in using other sequences presently identified as belonging to *C.rubrisquamis*, as these are likely to be *C.finifenmaa*.

#### Material examined.

***Cirrhilabrusrubrisquamis***: ROM 35932, 40.6 mm SL, juvenile holotype, Isla Fouquet, Peros Banhos Atoll, Chagos Archipelago**. *Cirrhilabrusfinifenmaa***: BPBM 33094, 69.2 mm SL, male holotype, east coast of Vilingili Island, North Malé Atoll, Maldives, 52 m, collected by John E. Randall with quinaldine and spear, 23–25 March 1988; BPBM 41385 (formerly BPBM 33094) (5), 35.8–70.5 mm SL, 1 juvenile, 2 females, 2 males, paratypes, collected with male holotype (Fig. 2C, E); AMS I.50058-001 (3), 54.0–69.1 mm SL, 2 males, 1 female, paratypes, Hulhumalé Island, North Malé Atoll, Maldives, 35–40 m, collected by Ibrahim Rasheed with hand nets, 15 August 2021; CAS-ICH 247311 (ex AMS I.50058-001), 57.2 mm SL, female, collected with AMS I.50058-001; ZRC 62259, 76.7 mm SL, male paratype, aquarium specimen from Maldives (Figs 2G, 3); ZRC 62249, 54.2 mm SL, male paratype, aquarium specimen from Maldives; ZRC 61604, 47.9 mm SL, female paratype, aquarium specimen from Maldives; ***Cirrhilabruswakanda***: CAS 246395, 70.3 mm SL, male holotype, east coast of Zanzibar, Tanzania, Africa, 75 m, collected by H.T. Pinheiro, B. Shepherd, and L.A. Rocha, 14 December 2018; CAS 246396, 56.8 mm SL, female paratype, east coast of Zanzibar, Tanzania, Africa, 70 m, 07 December 2018; CAS 246397, 61.3 mm SL, male paratype, same data as holotype; CAS 246398, 57.4 mm SL, female paratype, same data as holotype; CAS 246399, 54.3 mm SL, female paratype, same data as holotype.

## ﻿Taxonomic status of *Cirrhilabrusrubrisquamis* and comparisons to related species

In their description of *Cirrhilabrusrubrisquamis*, Randall and Emery (1983) could not reliably determine the sex of the holotype due to the absence of mature gonads. They note the presence of a blunt genital papilla (which we confirm), and while suggestive of the holotype being female, is difficult to confirm due to the small size of the specimen. Distinction between juveniles and females for many species of *Cirrhilabrus* on the basis of external characters is often difficult, with few reliable coloration characters separating these life stages. Depending on the species, *Cirrhilabrus* may possess one or more of the following coloration characters that feature prominently in the juvenile phase, but later disappearing or attenuating with maturity: presence of several fine, thread-like stripes interspersed with white spots on the upper half of the body; presence of a large white spot on the tip of the snout (Kuiter 2010); presence of a black caudal peduncular spot. The color photograph of the holotype in Randall and Emery (1983; also included here in Fig. 1) shows the presence of fine, thread-like stripes interspersed with white spots situated dorsally, below the dorsal fin origin. This combination of typical juvenile coloration pattern and the absence of mature gonads strongly suggests that the holotype is a juvenile.

*Cirrhilabrusrubrisquamis* is most closely related to *C.apterygia* from Western Australia, *C.blatteus* from the Red Sea, the newly described *C.finifenmaa* from the Maldives, *C.sanguineus* from Mauritius, and *C.wakanda* from East Africa. The six species form a clade of Indian Ocean species that belong to a larger group of fairy wrasses known as the *C.jordani* species complex, with species distributed across the Indo-Pacific (Tea et al. 2021b). Of the aforementioned species, the newly described *C.finifenmaa* is most distinct in having a rounded caudal fin (vs. lanceolate in *C.apterygia*, *C.blatteus*, *C.sanguineus*, and *C.wakanda* [terminal males not known in *C.rubrisquamis*; but see below]), in having purple-red crosshatch markings on its opercle, chest, isthmus, and anterior body, and in having unmarked scales on the posterior two-thirds of its body. On the basis of meristic, morphometric, and morphological details, *C.finifenmaa* further differs from: *C.apterygia* in having pelvic fins (absent in *C.apterygia*); *C.blatteus* in having a longer snout (8.1–9.5% SL vs. 7.5–8.1% SL) and a longer preanal length (60.1–67.3% SL vs. 52.1–62.5% SL); *C.sanguineus* in having a longer snout (8.1–9.5% SL vs. 7.7–8.7% SL); and *C.wakanda* in having a larger orbit (8.8–11.7% SL vs. 6.6–9.0% SL) and a taller dorsal fin (eight to ninth soft dorsal-fin ray longest, 14.0–15.5% SL vs. sixth longest, 11.9–14.3% SL).

Due to the lack of adequate material and muddled taxonomy of *C.rubrisquamis*, appropriate comparisons to related species of *Cirrhilabrus* is difficult. In particular, open questions remain regarding the taxonomic status of *C.rubrisquamis* and *C.wakanda*. Tea et al. (2019) described *C.wakanda* based on material collected from Zanzibar, Tanzania. They compared their new species with related *Cirrhilabrus* from the Indian Ocean, including *C.rubrisquamis*. Although their comparative data for *C.rubrisquamis* included published data of the holotype from Chagos, it also included published data from Randall’s (1995) Maldivian specimens (= *C.finifenmaa*). While the taxonomy of *C.rubrisquamis* sensu lato is here resolved with the description of *C.finifenmaa* from Maldives, comparative data taken from the *C.rubrisquamis* holotype and Randall’s Maldivian material (= *C.finifenmaa*) in the description of *C.wakanda* resulted in a potentially new problem.

Both *C.rubrisquamis* and *C.wakanda* are most similar on the basis of coloration patterns, in particular the presence of purple chain-link markings across the dorsal two-thirds of the body. Both species also share similar morphometric details (Table 1); however, this is not uncommon in closely related species of *Cirrhilabrus*, where identification is most reliable through comparison of terminal males (Tea et al. 2021a). Comparison of live coloration details between the smallest (54.3 mm SL) paratype of *C.wakanda* from Tea et al. (2019) and the 40.6 mm SL holotype of *C.rubrisquamis* reveal several minor coloration differences, such as *C.rubrisquamis* having a yellow dorsal fin (vs. fuchsia in *C.wakanda*), and a hyaline caudal fin with yellow and blue vermiculation (vs. caudal fin with a magenta chevron marking in *C.wakanda*). The holotype of *C.rubrisquamis* further differs from *C.wakanda* in having fewer pored lateral line scales (21–22 vs. 24–28). However, since *C.rubrisquamis* is known only from the juvenile holotype, it is unclear whether these differences represent distinct characters or ontogenetic variation.

**Figure 5.**
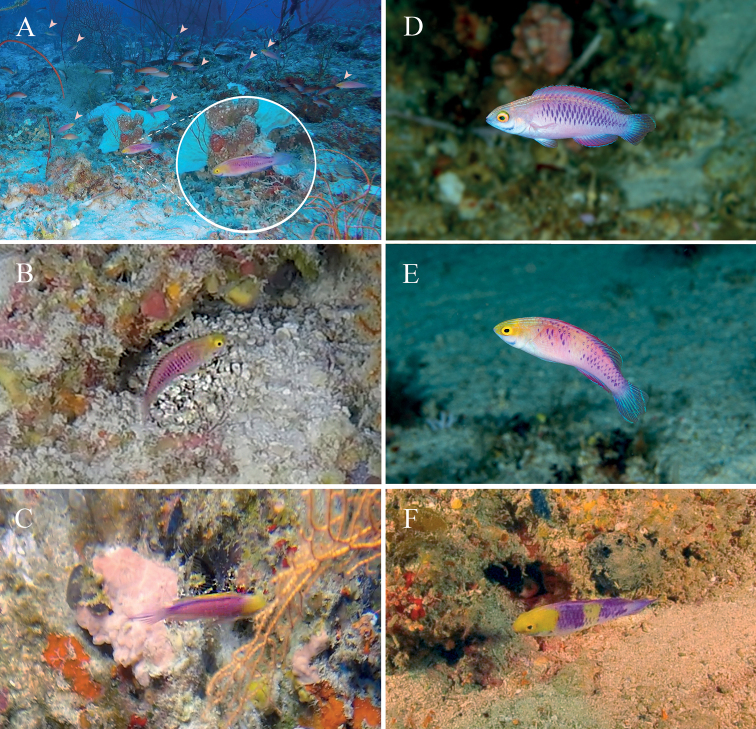
Underwater photographs of *Cirrhilabrus* from the Western Indian Ocean **A** harem of putative *Cirrhilabrusrubrisquamis*. Male depicted in circular inset, females by white arrowheads **B** putative female *C.rubrisquamis***C** putative terminal male *C.rubrisquamis*; Photographs taken from video footage provided by K Howell, N Foster, and C Diaz from the University of Plymouth Research Expedition to Egmont Atoll and Sandes Seamount in the Chagos Archipelago, 60–70 m **D** female *C.wakanda*, underwater photograph from Zanzibar, Tanzania, Africa, 75 m **E** male *C.wakanda*, underwater photograph from Zanzibar, Tanzania, Africa, 75 m **F** terminal male *C.wakanda*, underwater photograph from Moyette, off the coast of Mozambique, 100 m. Note fuchsia dorsal fin in all individuals and pale yellowish saddle in males. Photographs by LR (**D, E**) and P Plantard (**F**).

Recent underwater ROV explorations by the University of Plymouth Research Expedition to Egmont Atoll and Sandes Seamount in the Chagos Archipelago recovered in situ footage of fairy wrasses resembling *C.rubrisquamis* taken at depths between 60–70 m (Fig. 5A–C). Given the close proximity to Peros Banhos Atoll (also in the Chagos Archipelago), we strongly suspect these to be the nominal *C.rubrisquamis*. Examination of live coloration details of putative females (Fig. 5B) taken from the video footage agree well with the juvenile holotype of *C.rubrisquamis* and females of *C.wakanda* (Fig. 5D), particularly in having similar purple chain-link markings. The putative males (Fig. 5A, C) are similar in appearance to the East African *C.wakanda* (Fig. 5E, F), differing only slightly in having a yellow dorsal fin (Fig. 5C; vs. fuchsia in *C.wakanda*) and in lacking the yellowish saddle in the middle of the body (Fig. 5E, F). Given the similar live coloration of *C.rubrisquamis* and *C.wakanda*, it is possible that the two nominal species are conspecific. Observable differences (or potential lack thereof) in live coloration details however may be attributable to differences in size of the specimens, poor video resolution, and differences in underwater light properties.

The distribution of *C.wakanda* in East Africa and *C.rubrisquamis* in the Chagos Archipelago presents a unique pattern of biogeography shared by few congeneric *Cirrhilabrus*. Within the Indian Ocean, there appears to be faunal distinction between the Chagos Archipelago from the rest of the Western and Eastern Indian Ocean in some, but not all taxa. This biogeographic connectivity has been reported for corals (Obura 2012), *Acanthaster* sea stars (Haszprunar et al. 2017), and coconut crabs (Sheppard et al. 2013), and amongst fishes, at least 54 species are known to have a Western Indian Ocean distribution encompassing the Chagos Archipelago (Winterbottom and Anderson 1997). Pertinent examples of allopatric species conforming to this biogeographic pattern include the labrids *Halichoeresiridis* (East Africa to Chagos) and *H.leucoxanthus* (Maldives to Java), and two apparent species of *Dascyllus*, currently confused as *D.carneus* (see Gill and Kemp 2002). Marine shore fish endemism is particularly low in the Chagos Archipelago (Winterbottom et al. 1989), but with few notable examples (e.g., *Amphiprionchagosensis*). Without additional material of *C.rubrisquamis* of appropriate sizes from the type locality however, we are unable to reconcile the taxonomic status of both species, and whether or not *C.rubrisquamis* and *C.wakanda* represent closely related allopatric species, or whether they represent a single widespread conspecific taxon. Until such material from Chagos becomes available, we refrain from placing *C.wakanda* in the synonymy of *C.rubrisquamis*, and provisionally regard both species as valid.

## Supplementary Material

XML Treatment for Cirrhilabrus rubrisquamis

XML Treatment for Cirrhilabrus finifenmaa
